# Regional survey into trainee experience of core psychotherapy training

**DOI:** 10.1192/bjo.2021.386

**Published:** 2021-06-18

**Authors:** Lauren Evans, Georgia Belam

**Affiliations:** 1 South West London and St George's Mental Health NHS Trust; 2 Surrey and Borders Partnership NHS Foundation Trust

## Abstract

**Aims:**

This project aims to assess the experience of psychiatry core trainees who have undertaken core psychotherapy training (CPT), to identify what is experienced positively and potential areas of improvement.

**Background:**

Psychotherapy is an necessary part of core psychiatry training, requiring one short and one long case to complete core training.

**Method:**

An anonymous online survey was drafted, containing both qualitative and quantitative questions, to assess trainees experiences of CPT. It was circulated via Trust email (locally) and Twitter (nationally).

**Result:**

A total of 35 responses were received: 21 core trainees, 12 higher trainees, 1 consultant, and 1 staff grade doctor. 6 respondents had completed a short case only; 2 a long case only; 25 both; and 2 neither.

Confidence in psychotherapy knowledge was rated on a 1–5 scale (1: significantly below average to 5: significantly above average). Theoretical knowledge improved from a 2.57 average before CPT to 3.63 following, and clinical application improved from 2.43 before to 3.66 following.

Knowledge prior to delivering CPT was most commonly obtained from Balint group (71.4% of respondents) and MRCPsych courses (65.7%).

The main barriers to obtaining psychotherapy experience were: accessing supervision (60.0% of respondents); not enough patients (53.3%); and a lack of guidelines on accessing supervision and patients (43.3%). Additionally, getting time away from day jobs was identified as a concern, particularly among LTFT trainees.

Important learning points from CPT identified by trainees were: knowledge of psychoanalytic concepts, such as transference and counter-transference; differences between the theoretical models; an alternative approach to formulation; and how these skills can be useful in all clinical encounters, such as maintaining rapport, boundaries, and time-keeping. The useful role of supervision was also highlighted.

**Conclusion:**

This project serves as an introductory look into how trainees view their experience of CPT, and potential areas for improvement.

Themes for improvement, arising from qualitative responses, are: clear reading list, including introductory materials; clear guidelines at induction, including supervisor contact details; improved access to supervision; patients to be allocated; protected time for psychotherapy, with extra support for LTFTs; shadowing; increased choice of modality; and more formal teaching on psychotherapy. These are key areas to be targeted to improve the trainee experience, particularly for those who risk delays in their training.

